# Question No. 23

**Published:** 1890-06-28

**Authors:** 


### Passing Topics.

### QUESTION No. 23.

It would be interesting to know how the several hospitals have decided to reply to Question No. 23 and last in the series of inquiries put by the Council of the Hospital Saturday Fund to the governing bodies of institutions aspiring to participate in the collection for the current year. It would be still more interesting to learn something of the discussions which must have preceded the decision.

That there has been considerable hesitation we may take for granted. Put to individual hospitals, the question is a puzzling one; considered, if it could be, collectively, the reply might be foretold with safety, and the case seems to afford a very fair illustration of the occasional necessity for common action. We may assume that there are but three ways of dealing with the question, which runs thus: "23. Are you willing to give to the Hospital Saturday Fund, in return for grants, 'letters' in the same proportion as you give to ordinary subscribers?" We may say "Yes" or "No," or we may refuse to reply at all. It may be considered certain that every institution which replies "Yes" will do so with more or less bad grace, and in spite of the feeling that it is being imposed upon; that few will have the courage to say "No" outright, and that some will attempt to evade the dilemma in which they are placed, by adopting the third alternative. As to any hospital whatever agreeing that the demand made is a righteous one, to be granted with satisfaction, we may dismiss the notion as absurd. The Sunday Fund authorities, it is well known, have aroused adverse feeling in some quarters by asking for one-half the number of letters customary in the case of an ordinary subscriber. Yet the Sunday Fund grants are in the nature of philanthropic offerings, to a degree unapproached by the accumulated pence of the Saturday Fund which are collected, ostensibly, at any rate, from the classes making direct use of hospitals, and represent, as is well known, less than one and a-half per cent. of a total sum expended almost wholly upon the givers.

Consider what is meant by "letters in the same proportion as you give to ordinary subscribers." Two hundred recommendations, or thereabouts, for every £100 granted! It is no sufficient reply to say that one contributor's money is as good as another's, and what is conceded in the one case may safely be given in the other. That is to beg the whole question. Subscribers' "privileges" are questionable things at the best of times, but in the present case the objection lies in the radical and essential difference between the two classes of contributions. The money of the Saturday Fund is composed of minute offerings from manifold persons, every one of whom is a hospital patient *in posse*, and the hospitals must be prepared not for the infrequent and merely nominal exercise of a privilege, but for an absolute, deliberate, and systematic assertion of whatever rights are secured.

It is easy then to appraise the bargain the astute authorities of the Saturday Fund are seeking to make. It is nothing less than that the hospitals should return, for every £100 granted them, food, medicine, and nursing, to the value of, say, £250, plus the unrealised value of such trifling items as the physician's or surgeon's skill and attention, and all honorary services.

Hospitals will no doubt be found willing to put the Saturday Fund grant into their treasuries without weighing the consequences, but the briefest reflection must convince any impartial person that the Council of the Saturday Fund are throwing themselves open to the charge that they are endeavouring to take advantage of the poverty of our medical charities to exact an unfair and immoral compact which the victims will only be able to act up to by evading other and more legitimate obligations.

Agricola.

